# Predictive Factors of Radiation-Induced Changes Following Single-Session Gamma Knife Radiosurgery for Arteriovenous Malformations

**DOI:** 10.3390/jcm10102186

**Published:** 2021-05-19

**Authors:** Myung Ji Kim, Kyung Won Chang, So Hee Park, Won Seok Chang, Jong Hee Chang, Jin Woo Chang, Hyun Ho Jung

**Affiliations:** 1 Department of Neurosurgery, Korea University Ansan Hospital, Korea University College of Medicine, 123 Jeokgeum-ro, Danwon-gu, Ansan, Gyeonggi-do 15355, Korea; kmj868686@gmail.com; 2 Department of Neurosurgery, Brain Research Institute, Yonsei University College of Medicine, Yonsei-ro 50-1, Seodamungu, Seoul 03722, Korea; KWCHANG90@yuhs.ac (K.W.C.); SHPARK123@yuhs.ac (S.H.P.); CHANGWS0716@yuhs.ac (W.S.C.); CHANGJH@yuhs.ac (J.H.C.); JCHANG@yuhs.ac (J.W.C.)

**Keywords:** adverse radiation effect (ARE), arteriovenous malformation (AVM), cerebrovascular disease (CVD), gamma knife radiosurgery (GKRS), stereotactic radiosurgery (SRS), radiation-induced change (RIC)

## Abstract

We evaluated for possible predictors of radiation-induced changes (RICs) after gamma knife radiosurgery (GKRS) for arteriovenous malformations (AVMs). We identified the nidal component within AVMs to analyze the correlation between the volume of brain parenchyma within the 50% isodose line (IDL) and RICs. We retrospectively reviewed patients with AVMs who underwent a single-session of GKRS at our institution between 2007 and 2017 with at least a 2-year minimum follow-up. Follow-up magnetic resonance images were evaluated for newly developed T2 signal changes and the proportions of nidus and intervening parenchyma were quantified. A total of 180 AVM patients (98 males and 82 females) with a median age of 34 years were included in the present study. The overall obliteration rate was 67.8%. The median target volume was 3.65 cc. The median nidus and parenchyma volumes within the 50% IDL were 1.54 cc and 2.41 cc, respectively. RICs were identified in 79 of the 180 patients (43.9%). AVMs associated with previous hemorrhages showed a significant inverse correlation with RICs. In a multivariate analysis, RICs were associated with a higher proportion of brain parenchyma within the 50% IDL (hazard ratio (HR) 169.033; *p* < 0.001) and inversely correlated with the proportion of nidus volume within the 50% IDL (HR 0.006; *p* < 0.001). Our study identified that a greater proportion of brain tissue between the nidus within the 50% IDL was significantly correlated with RICs. Nidus angioarchitectural complexity and the absence of a prior hemorrhage were also associated with RICs. The identification of possible predictors of RICs could facilitate radiosurgical planning and treatment decisions as well as the planning of appropriate follow-up after GKRS; this could minimize the risk of RICs, which would be particularly beneficial for the treatment of incidentally found asymptomatic AVMs.

## 1. Introduction

Gamma knife radiosurgery (GKRS) is a treatment option for cerebral arteriovenous malformations (AVMs) and is especially effective for AVMs located in deep or eloquent areas of the brain [1,2]. However, there are several adverse effects associated with GKRS for AVMs, including brain edema, necrosis, delayed cyst formation, arterial stenosis, encapsulated hematoma, and hemorrhage after obliteration [3,4,5,6,7,8]. Radiation-induced changes (RICs) are the most common complication after GKRS for AVMs; they appear as T2-weighted hyperintensities on magnetic resonance imaging (MRI) and occur in 30–40% of patients [9,10,11,12,13,14,15]. Although most RICs are asymptomatic [10,12], the incidence of symptomatic RICs has been reported to be between 3.7% and 10.8% [12,16,17]. While most symptomatic RICs are transient and can be medically manageable, a few patients (1–5.1%) develop a permanent neurologic deficit [12,14,16,17]. Previous studies have suggested that AVM location, nidus volume, radiation dose, and the brain volume included in the 12 Gy dose are predictive factors for RICs [11,12,13,14,18]. Importantly, AVMs contain no pathological tissue and the delivered dose is concentrated on a confined target volume [19]; thus, it is not possible to exclude brain parenchyma from radiation exposure, which can lead to radiation injury to the intervening brain parenchyma within the isodose line (IDL) [20]. The underlying mechanism of RICs remains to be elucidated. Although target volume has been suggested as an important predictor of RICs [10,11,14,17,21,22], the incidence of RICs in large AVMs is not consistently higher than that of smaller AVMs. AVMs that are widespread and intermingled with brain parenchyma are more likely to receive a higher dose of radiation than AVMs with compact vascular structures and little intervening brain tissue [20]. We hypothesize that RICs might be associated with injuries to the brain parenchyma. In the present study, we evaluated possible predictive factors associated with the development of RICs after GKRS for AVMs. We attempted to identify nidal components within AVMs to analyze the correlation between the volume of brain parenchyma within the 50% IDL and RICs.

## 2. Materials and Methods

### 2.1. Patient Characteristics

A database of 453 patients with AVMs, who underwent GKRS between January 2007 and December 2017 in our center, was retrospectively reviewed. This study excluded patients who had less than 2 years of follow-up or who had been previously treated by repeat or volume-staged GKRS, resection or embolization in our center or at another institution; this was to allow us to evaluate the effect of a single-session of GKRS. All patients had at least 2 years of imaging follow-up that included thin-slice post-GKRS MRI sequences for volumetric analysis, and cerebral angiography follow-up studies at our hospital available for analysis. Patient clinical data were reviewed, including demographics, imaging findings prior to GKRS, radiosurgical parameters, and follow-up images. AVMs were classified using the Spetzler–Martin (SM) grade, Virginia Radiosurgery AVM Scale (VRAS), and Pollock–Flickinger score [23]. This study obtained full ethical approval from our Institutional Review Board (IRB).

### 2.2. Gamma Knife Radiosurgery

The procedure for GKRS has been described previously [23,24,25]. In brief, all patients underwent stereotactic frame placement and then neuroimaging including MRI and digital subtraction angiography (DSA) [23]. Radiosurgery was performed using the Leksell Gamma Knife Unit Model C between 2007 and 2008, and the Perfexion Model (Elekta AB, Elekta Company, Stockholm, Sweden) between 2009 and 2017. Stereotactic MRI with T1-weighted contrast-enhanced and T2-weighted imaging sequences, as well as DSA using Leksell GammaPlan (Elekta AB, Stockholm, Sweden), were used to delineate the AVM nidus. A neurosurgeon and a medical physicist prescribed the radiation dose based on the location of the AVM nidus and the calculated lesion volume [23].

### 2.3. Neuroimaging Follow-Up and Outcome Assessment

Following GKRS, all patients were evaluated clinically and MRI and MR angiography (MRA) with time-of-flight (TOF) studies were performed at 6-month intervals for the first 2 years, then annually thereafter. It was suggested that a cerebral angiogram be performed once the nidus was deemed obliterated on MRI/MRA [23]. RICs were identified as newly developed perinidal hyperintensities on T2-weighted MRI and were classified according to the grading system proposed by Yen et al. [7]. Symptomatic RICs were defined as RICs associated with any newly developed headache, seizure, or neurologic deficit. The absence of nidus filling on the angiography was defined as total obliteration of the AVM. If there was no flow void on MRI and vascular filling on MRA in case the patient did not have an angiogram, the AVM was considered obliterated [23,24]. The incidence of latent period hemorrhage and delayed cyst formation were assessed using MRI throughout the follow-up period.

### 2.4. Volumetric Analysis

**Figure 1:**
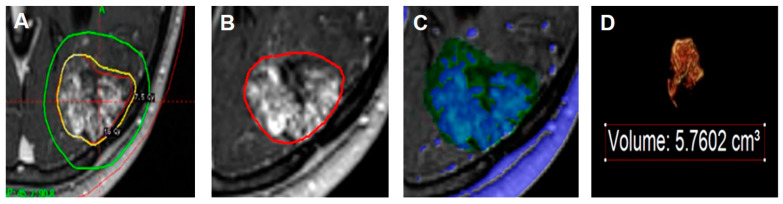
The process of the volumetric analysis. (**A**) The red line represents the target volume and the yellow line represents the 50% isodose line (IDL) in the Leksell GammaPlan. (**B**) The 50% IDL (red) is demarcated manually using Aquarius and defined as the measured volume within the 50% IDL. (**C**) The nidus volume within the 50% IDL (blue in green background) is calculated by adjusting the contrast threshold of the selected volume within the 50% IDL (green background). (**D**) The parenchyma volume within the 50% IDL is determined by subtraction of the nidus volume from the measured volume within the 50% IDL.

The nidus volume was initially measured using the Leksell GammaPlan at the time of treatment and was defined as the target volume which might contain intermingled normal brain parenchyma as well as the nidus. The contrast-enhancing portion of the AVM, excluding any draining veins or arterial feeders, was defined as the nidus [26]. The volume within the 50% IDL, defined as the measured volume within the 50% IDL, was measured manually on T1-weighted contrast-enhanced imaging using Aquarius (version 4.4.13.P6, TeraRecon, Durham, NC, USA). The nidus volume within the 50% IDL was calculated by adjusting the contrast threshold of the selected volume within the 50% IDL, and the parenchyma volume within the 50% IDL was determined by subtraction of the nidus volume from the measured volume within the 50% IDL. Figure 1 presents an example of the volumetric analysis used in the present study.

### 2.5. Statistical Analysis

None of the continuous variables satisfied normality when tested using the Shapiro–Wilk test and were presented as medians and range (Q1: cumulative percentage of 25%, Q3: cumulative percentage of 75%). Categorical variables were presented as frequency and percentages. The chi-square test and Fisher’s exact test were used to examine differences between the RIC and non-RIC groups. All endpoints including RICs, obliteration, latent period hemorrhage, and delayed cyst formation were analyzed using the Kaplan–Meier analysis with log-rank tests. Univariate and multivariate analyses were performed using the Cox proportional hazards regression model to analyze the predictive factors for RICs after GKRS for AVMs. The hazard ratio (HR) and 95% confidence interval (CI) were calculated. Logistic regression modeling was used to analyze the predictive factors for RIC grading categories by assessing odds ratios (OR). Grading scales were not included in the multivariate analysis because of multicolinearity. Contal and O’Quigely’s method was used to find the cut off value of the proportion of the parenchyma within the 50% IDL where the Kaplan–Meier curve of RICs was maximal (i.e., the point where the log-rank test was most significant). A *p* value of less than 0.05 suggested statistical significance. All analyses were performed using statistical software (SAS version 9.4, SAS Inc., Cary, NC, USA; and R package, version 3.6.3).

## 3. Results

### 3.1. Patient Demographics, AVM Characteristics, and Radiosurgical Parameters

Among 453 GKRS procedures for AVMs, 126 were repeated sessions of GKRS; therefore, a total of 327 patients underwent single-session GKRS. Fifty-eight patients were excluded because their follow-up periods were less than 2 years and they did not have follow-up imaging studies. Eighty-nine patients who underwent embolization or surgical resection prior to receiving GKRS were also excluded. In total, 180 patients were enrolled in this study. Table 1 presents details about the patient demographics, AVM characteristics, and radiosurgical parameters. The median measured volume within the 50% IDL was slightly larger than the median target volume (4.18 cc vs. 3.65 cc). The median nidus volume within the 50% IDL was 1.54 cc and the median parenchyma volume within the 50% IDL was 2.41 cc (Table 1).

### 3.2. Treatment Outcomes

**Figure 2:**
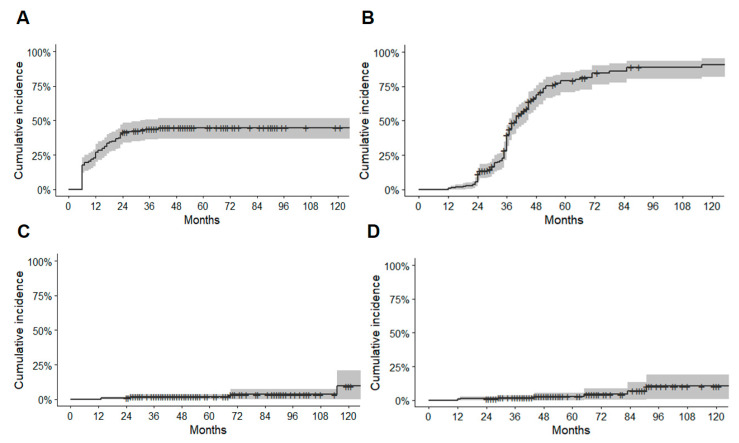
Kaplan–Meier curves. (**A**) Kaplan–Meier curve for radiation-induced changes (RICs). (**B**) Kaplan–Meier curve for obliteration. (**C**) Kaplan–Meier curve for latent period hemorrhage. (**D**) Kaplan–Meier curve for delayed cyst formation.

Seventy-nine patients (43.9%) developed RICs following GKRS, with 38 (48.1%) classified as Grade I, 37 (46.8%) as Grade II, and 4 (5.1%) as Grade III. The median duration from GKRS to the development of RICs was 11 months (range 6–40 months). The imaging changes vanished entirely within 6–89 months following the development of RICs. The RICs were symptomatic in 20 patients (25.3%) among the patients who developed RICs during the follow-up period, resulting in an overall incidence of symptomatic RICs of 11.1%. Two of these patients had severe headaches and nausea, three had visual field deficits, one had dysarthria, one had paresthesia, seven had hemiparesis, and six had new-onset seizures after GKRS. Most of these symptoms were reversible. Seven patients (8.9%) had permanent deficits following the development of RICs, resulting in an overall incidence of permanent RIC of 3.9%. Three of these patients underwent surgery for radiation necrosis (Table 1). The Kaplan–Meier curve for RICs is shown in Figure 2A. In the present study, MRI/MRA or angiographic obliteration after GKRS was achieved in 112 patients (67.8%; Table 1). The complete absence of nidus filling on an angiography was identified in 108 patients (88.5%) and the absence of flow void and vascular filling on MRI/MRA in 14 patients (11.5%). The Kaplan–Meier curve for obliteration is shown in Figure 2B. Five patients (2.8%) experienced latent period hemorrhage and seven patients (3.9%) experience delayed cyst formation after GKRS (Table 1). The Kaplan–Meier curves shown in Figure 2C,D are for latent period hemorrhage and delayed cyst formation, respectively.

### 3.3. Radiation-Induced Change

Table 2 demonstrates the difference between the RIC and non-RIC groups in relation to baseline demographics, AVM characteristics, radiosurgical parameters, and treatment outcomes. Statistically significant differences were found between the two groups in terms of target volume, measured volume, nidus volume, parenchyma volume within the 50% IDL, VRAS, Pollock–Flickinger score, SM grade distribution, history of ruptured AVMs, and incidence of delayed cyst formation after GKRS (Table 2). Although the RIC group included more AVMs with a larger target volume, measured volume, nidus volume, and parenchyma volume within the 50% IDL compared with the non-RIC group, the proportion of parenchyma volume within the 50% IDL (P/M) was significantly higher than in the non-RIC group. The non-RIC group was significantly more likely to report a history of hemorrhage due to a ruptured AVM before GKRS than the RIC group (55.5% vs. 29.1%). No statistically significant differences in marginal dose, obliteration rates, and latent period hemorrhage after GKRS were found between the two groups.

The Cox regression model evaluating factors related to time to RICs is shown in Table 3. In the univariate analysis, those with a higher target volume, measured volume within the 50% IDL, nidus volume within the 50% IDL, parenchyma volume within the 50% IDL, and higher VRAS, Pollock–Flickinger score, and SM grade indicated a significantly higher risk of developing RICs. Those with a higher proportion of nidus volume within the 50% IDL showed a significant inverse correlation with RICs, while those with a higher proportion of parenchyma volume within the 50% IDL showed a significantly higher risk of developing RICs. A negative history of prior hemorrhage was correlated with a higher risk of developing RICs. In the multivariate analysis, grading scales were not included and the nidus volume and parenchyma volume within the 50% IDL were selected except for variables such as the target volume and measured volume within the 50% IDL, which caused multicollinearity. Predictors for RICs were a higher parenchyma volume within the 50% IDL (HR = 1.042, *p* = 0.0140), low nidus volume within the 50% IDL (HR = 0.878, *p* = 0.0139), and no previous history of ruptured AVMs (HR = 0.312, *p* <0.0001) in the multivariate analysis (Table 3). AVMs associated with RICs had significantly greater proportions of intermingling brain parenchyma between the nidal component compared to AVMs not associated with RICs.

**Figure 3:**
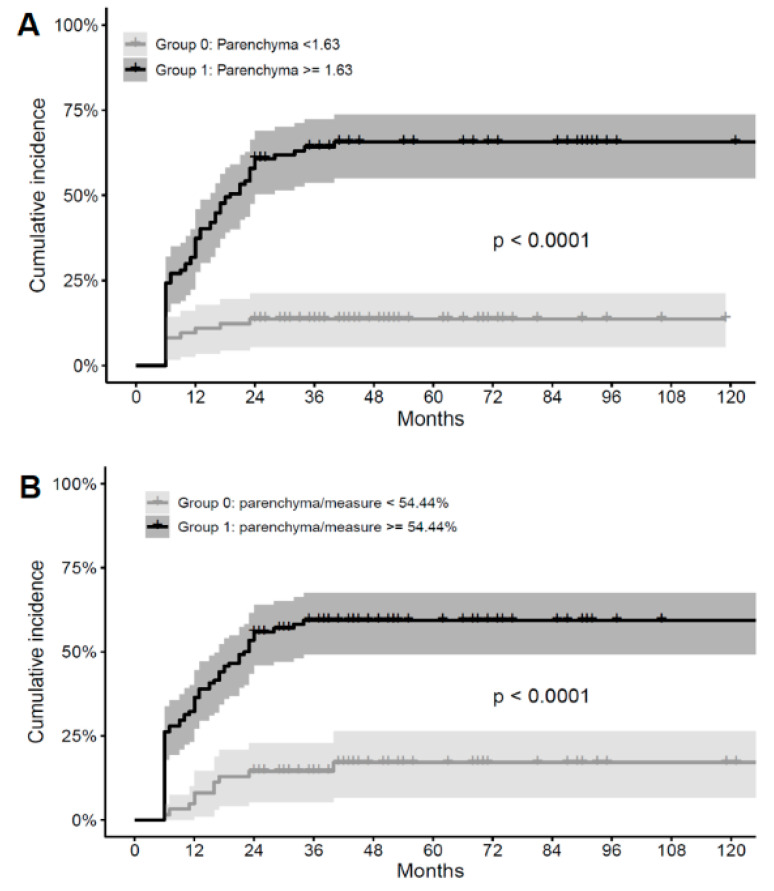
The cut off value of parenchyma where the Kaplan–Meier curve of RICs is most maximized. (**A**) The cut off value of parenchyma within the 50% isodose line (IDL) where the Kaplan–Meier curve of RICs is maximal: 1.63 cc. (**B**) The cut off value of the proportion of parenchyma within the 50% IDL where the Kaplan–Meier curve of RICs is most maximized: 54.4%.

The logistic regression model for factors regarding RIC grade is shown in Table 4. The logistic regression analysis demonstrated a similar result to the Cox regression analysis. In terms of the grade of RICs, AVMs with a higher parenchyma volume within the 50% IDL (OR = 1.095, *p* = 0.0045) were more likely to present with a higher grade of RICs; AVMs with a higher nidus volume within the 50% IDL (OR = 0.842, *p* = 0.0114) and a previous history of rupture-associated hemorrhages (OR = 0.217, *p* <0.0001) were more likely to present with a lower grade of RICs in the multivariate analysis (Table 4). Using Contal and O’Quigely’s method to determine a significant cut off value for parenchyma volume within the 50% IDL for RICs, we found a significant threshold of 1.63 cm^3^ (Figure 3A), with a significant proportion of 54.4% within the 50% IDL (Figure 3B).

## 4. Discussion

In the present study, we investigated possible predictive factors for RICs following single-session GKRS for AVMs and attempted to identify correlative factors between the parenchyma volume within the 50% IDL and the development of RICs. We focused on imaging changes after GKRS and a volumetric analysis with or without newly developed neurological symptoms.

The incidence of RICs observed following GKRS treatment of AVMs is higher than that seen following GKRS treatment of tumors, which demonstrates that RICs do not only occur due to focal radiation injury. Although the pathophysiology of RICs remains to be elucidated, several mechanisms have been implicated. RICs have been thought to be the result of blood–brain barrier disruption, resulting in brain edema [10,14,27]. RICs may be caused not only by vascular endothelial damage within the nidus but also by ischemic insults within normal brain tissue between the nidus.

The predictive factors for RIC development following GKRS for AVMs include marginal dose, target volume, location, AVM angioarchitecture, history of AVM rupture, prior resection, and embolization; among these factors, the marginal dose and target volume have been suggested to be important predictive factors of the development of RICs.

In the present study, we did not identify a significant correlation between the marginal dose and RICs. However, previous studies have suggested that a higher marginal dose is significantly correlated with a higher incidence of RICs, based on the long-term results of lower-dose GKRS for AVM [3,4,5,6,10,28,29]. The median marginal dose was 16 Gy (15,17) (Table 1) and ranged from 12 to 20 Gy. Although there are no strict rules for prescribing the optimal radiation dose, an optimal marginal dose was administered between 16 and 18 Gy according to the location, volume, and age in our center. For AVMs located in eloquent areas, large AVMs, or pediatric patients, a marginal dose lower than 16 Gy was prescribed, and for very small AVMs, up to 20 Gy were administered. Furthermore, 123 patients, 68.3% of the total, were treated with a marginal dose between 16 and 18. There was not much variation in the marginal dose, which could be one possible explanation as to why it did not appear as a predictive factor for RIC. In the current study, patients that underwent repeated GKRS were excluded in order to evaluate the effect of single-session GKRS on the development of RICs. From the perspective that the occurrence of RICs is dose-dependent, repeated radiosurgery has been suggested to be a risk factor for RICs [30].

Previous studies have proposed that a larger target volume is correlated with increased RICs [7,12,13,16,17,22,29], which is consistent with the results of our univariate analysis (Table 3). Han et al. suggested that a medium AVM (4–14 cm3) was significantly related to RICs instead of the AVM volume itself in 2008 [17], but Hayhurst et al. proposed a significant target volume threshold of 4 cm3 in 2010 [22].

Furthermore, the relationship between target volume and the development of RICs has been reported to be dose-dependent [11]. Previous publications have suggested that the brain volume included in the 12 Gy volume is predictive of RICs following radiosurgery for AVMs. A larger 12 Gy volume has been correlated with a higher risk of symptomatic RICs [4,11,12,31]. Another study proposed that 8-, 10-, and 12-Gy-Volumes, and a mean dose to a specified volume were associated with disruption of the blood–brain barrier following radiosurgery for AVMs, which led to the development of RICs [32]. The dose–volume relationship is without doubt one of the most significant predictors for the development of RICs.

In the current study, we analyzed not only the target volume but also attempted to measure the nidus and parenchyma volume within the 50% IDL and identify their correlation with the development of RICs. It is inevitable that radiosurgery for AVMs will induce radiation injury to the intermingled normal brain parenchyma between the nidus. It is also known that normal brain tissue is more vulnerable to radiation injury than pathologic structures such as brain tumors [33]. Recent studies with small numbers of patients have suggested that the relationship between AVM treatment and RICs might be correlated with the intervening nidal brain parenchyma [20,34]. In the present study, we calculated the nidus and parenchyma volumes within the 50% IDL and identified a significant relationship between the nidus and parenchyma volumes within the 50% IDL, and the development of RICs (Table 3) as well as the grade of the RICs (Table 4). A higher parenchyma volume within the 50% IDL (HR = 1.042, *p* = 0.0140) and lower nidus volume within the 50% IDL (HR = 0.878, *p* = 0.0139) were significant predictors for RICs in the multivariate analysis (Table 3). In addition, the RIC group had a greater proportion of parenchyma volume within the 50% IDL (P/M) than the non-RIC group (Table 2).

In the present study, we found that the RIC group had more complex AVM angioarchitectures (higher SM grade, Pollock–Flickinger score, and VRAS) than the non-RIC group (Table 2), and higher VRAS, SM grade, and Pollock–Flickinger score were associated with the development of RICs (Table 3) as well as the grade of the RICs (Table 4) in the univariate analysis, although the grading scales were not included in the multivariate analysis. These results are consistent with previous studies [35,36].

In the current analysis, we found a significant difference between the RIC and non-RIC groups with respect to the history of AVM ruptures before GKRS (Table 2), and a history of hemorrhages was inversely correlated with the occurrence of RICs (Table 3). Previous studies also proposed that a lack of prior AVM ruptures was significantly correlated with RICs [7,14,17,22]. Perinidal gliosis or fluid-filled spaces resulting from an AVM rupture might be protective against RICs [22,37]. Brain tissue that has not been injured from a ruptured AVM may be more sensitive to radiation exposure when GKRS is performed for unruptured and incidental AVMs.

Several prospective studies have reported that patients with unruptured AVMs who were managed medically had significantly better outcomes compared to those who underwent intervention [38,39,40]. However, GKRS for AVMs is still effective with acceptable complication rates, and advances in GKRS have resulted in even lower risks of morbidity and mortality associated with treatment. Physicians should be careful when deciding whether to treat unruptured AVMs, which proved to be one of the risk factors for RICs in the present study, especially in asymptomatic cases. We should make an effort to perform more elaborate radiosurgical planning that includes delineation of the target (nidus) and the prevention of radiation injury to the surrounding brain parenchyma; however, radiation injury to intermingled normal brain parenchyma is inevitable in particularly diffuse AVMs. Consideration of the proportion of the nidus and parenchyma within the prescription IDL will facilitate treatment decisions that alleviate RICs. Patients who develop RICs need longer imaging follow-ups to look for late RICs, including delayed cyst formation and radiation necrosis.

### Study Limitations

The major limitation of the current study is that it was a retrospective review of a single institution’s practice. Selection bias may have played an important role in patient selection because we excluded patients who had been previously treated by repeat or volume-staged GKRS, resection, or embolization to evaluate the effect of single-session GKRS. Combined embolization and/or volume-staged treatment approaches rather than single-session GKRS are reserved for larger AVMs with more complex angioarchitecture. The median target volume (3.65 cc) measured during the initial GKRS planning was bigger than the median nidus volume within the 50% IDL (1.45 cc), which was a more apparent difference than we expected. Previous studies reported the median proportion of the nidus was 31.3% of the target volume [34] and the percentage of brain tissue within the target varied from 31.13% to 70.85% [20]. These reveal that the target volume might contain more than expected normal brain parenchyma within the IDL. In addition, the 50% IDL was manually delineated for the volumetric analysis, which might thus be subject to a minor error. When adjusting the contrast threshold to determine the nidus volume within the 50% IDL, non-nidal components might include cerebrospinal fluid as well as brain parenchyma. Lastly, total obliteration of the AVM was confirmed on an angiography or MRI/MRA, which could lead to the overestimation of obliteration, although a majority of patients (88.5%) underwent angiography to confirm the complete absence of nidus filling.

## 5. Conclusions

This study found that greater proportions of brain tissue between the nidus within the 50% IDL were significantly correlated with the development of RICs. Nidus angioarchitectural complexity and the absence of a prior hemorrhage were also associated with the development of RICs. The prevalence of cyst formation was significantly higher in the RIC group than in the non-RIC group. Identification of the possible predictors of RICs could facilitate radiosurgical planning and enable us to make treatment decisions, plan appropriate follow-ups after GKRS, and eventually alleviate the risk of RICs; this would be especially beneficial when treating incidentally found asymptomatic AVMs.

## Figures and Tables

**Table 1:** Patient demographics, arteriovenous malformation (AVM) characteristics, radiosurgical parameters, and treatment outcomes.

	Total (*n* = 180)
Median age	34 (22, 46)
Sex	
Male	98 (54.4%)
Female	82 (45.6%)
Median FU, month	47 (35, 73.5)
Median marginal dose, Gy	16 (15,17)
Meadian target volume, cc	3.65 (1.50, 9.09)
Median measured volume within 50% isodense line, cc	4.18 (1.74, 10.17)
Median nidus volume within 50% isodense line, cc	1.54 (0.62, 4.19)
Median parenchyma volume within 50% isodense line, cc	2.41 (0.94, 5.94)
VRAS	
0	12 (6.7%)
1	38 (21.1%)
2	60 (33.3%)
3	57 (31.7%)
4	13 (7.2%)
Pollock–Flickinger score	
<1	59 (32.8%)
1.01–1.50	55 (30.6%)
1.51–2.00	35 (19.4%)
>2	31 (17.2%)
SM grade	
1	42 (23.3%)
2	78 (43.4%)
3	51 (28.3%)
4	9 (5.0%)
Lobar	143 (79.4%)
Deep	37 (20.6%)
Presence of Aneurysm (intranidal/flow-related)	27 (15.0%)
Initial ruptured AVMs	79 (43.9%)
Radiation induced changes (RICs)	79 (43.9%)
Grade 1	38 (48.1%)
Grade 2	37 (46.8%)
Grade 3	4 (5.1%)
Symptomatic RICs	20 (25.3%)
Permanent RICs	8 (8.9%)
Median duration from treatment to RICs, months	11 (6, 17)
Obliteration	122 (67.8%)
Angiography	108 (88.5%)
Magnetic resonance imaging and angiography	14 (11.5%)
Median duration from treatment to obliteration, months	36 (31, 45)
Latent period hemorrhage	5 (2.8%)
Delayed cyst formation	7 (3.9%)

FU = follow up, SM = Spetzler–Martin, VRAS = Virginia Radiosurgery AVM Scale. Descriptive statistics are presented as medians (Q1: cumulative percentage of 25%, Q3: cumulative percentage of 75%).

**Table 2:** Comparison of baseline demographic data, arteriovenous malformation (AVM) characteristics, radiosurgical parameters, and treatment outcomes between the radiation-induced change (RIC) group and non-RIC group.

	Non-RIC (*n* = 101)	RIC (*n* = 79)	*p*-Value
Median age	32 (16,45)	37 (25,47)	0.0576
Sex			0.7604
Male	56 (55.5%)	42 (53.2%)	
Female	45 (44.5%)	37 (46.8%)	
Median marginal dose, Gy	16 (15, 17)	16 (15, 17)	0.1079
Median target volume, cc	2.0 (0.9, 4.8)	6.5 (2.9, 13.5)	**<0.0001**
Median measured volume within 50% isodense line, cc	2.3 (1.0, 5.6)	6.7 (3.6, 15.8)	**<0.0001**
Median nidus volume within 50% isodense line, cc	0.9 (0.5, 2.6)	2.6 (1.1, 5.9)	**<0.0001**
Median parenchyma volume within 50% isodense line, cc	1.3 (0.6, 2.8)	4.7 (2.2, 9.8)	**<0.0001**
Median N/M (nidus/measured volume within 50% isodense line)	0.5 (0.3, 0.6)	0.3 (0.3, 0.4)	**<0.0001**
Median P/M (parenchyma/measured volume within 50% isodense line)	0.5 (0.4, 0.7)	0.7 (0.6, 0.8)	**<0.0001**
VRAS			**0.0075**
0	11 (10.9%)	1 (1.3%)	
1	24 (23.8%)	14 (17.7%)	
2	37 (36.6%)	23 (29.1%)	
3	23 (22.8%)	34 (43.0%)	
4	6 (5.9%)	7 (8.9%)	
Pollock–Flickinger score			**<0.0001**
< 1	48 (47.5%)	11 (13.9%)	
1.01–1.50	29 (28.7%)	26 (32.9%)	
1.51–2.00	15 (14.9%)	20 (25.3%)	
> 2	9 (8.9%)	22 (27.9%)	
SM grade			**0.0005**
1	32 (31.7%)	10 (12.7%)	
2	47 (46.5%)	31 (39.2%)	
3	20 (19.8%)	31 (39.2%)	
4	2 (2.0%)	7 (8.9%)	
Location			0.1151
Lobar	76 (75.3%)	67 (84.8%)	
Deep	25 (24.7%)	12 (15.2%)	
Presence of Aneurysm (intranidal/flow-related)			
Initial ruptured AVMs	56 (55.5%)	23 (29.1%)	**0.0004**
Obliteration	74 (73.3%)	48 (60.8%)	0.0748
Angiography	65 (87.8%)	43 (89.6%)	
Magnetic resonance imaging and angiography	9 (12.2%)	5 (10.4%)	
Latent period hemorrhage	2 (2.0%)	3 (3.8%)	0.6551
Delayed cyst formation	1 (1.0%)	6 (7.6%)	**0.0446**

SM = Spetzler–Martin, VRAS = Virginia Radiosurgery AVM Scale. Descriptive statistics are presented as medians (Q1: cumulative percentage of 25%, Q3: cumulative percentage of 75%). Boldface type indicates statistical significance (*p* < 0.05).

**Table 3:** Univariate and multivariate Cox proportional hazards regression analyses for predictors of radiation-induced change (RIC) after GKRS.

	Univariate Analysis			Multivariate Analysis		
Factors	HR	CI	*p*-Value	HR	CI	*p*-Value
Sex (female)	1.092	(0.702, 1.699)	0.6965			
Age	1.010	(0.998, 1.023)	0.1099			
Target volume	1.017	(1.007, 1.027)	**0.0008**			
Marginal dose	0.912	(0.779, 1.068)	0.2525			
Deep location	0.625	(0.338, 1.155)	0.1335			
Initial rupture	0.446	(0.274, 0.725)	**0.0011**	0.312	(0.179, 0.541)	**<0.0001**
SM grade	1.703	(1.317, 2.203)	**<0.0001**			
VRAS	1.441	(1.155, 1.798)	**0.0012**			
Pollock–Flickinger score	1.180	(1.074, 1.295)	**0.0005**			
Measured volume within 50% isodense	1.008	(1.002, 1.014)	**0.0138**			
Nidus volume within 50% isodense line	1.027	(1.000, 1.055)	**0.0475**	0.878	(0.792, 0.974)	**0.0139**
Parenchyma volume within 50% isodense line	1.011	(1.003, 1.019)	**0.0104**	1.042	(1.008, 1.077)	**0.0140**
Nidus/Measured volume within 50% isodense line	0.028	(0.006, 0.137)	**<0.0001**			
Parenchyma/Measured volume within 50% isodense line	35.323	(7.300, 170.921)	**<0.0001**			

HR = hazard ratio, CI = confidence interval, VRAS = Virginia Radiosurgery AVM Scale, GKRS = gamma knife radiosurgery, SM = Spetzler–Martin. Boldface type indicates statistical significance (*p* < 0.05). Grading scales were not included in the multivariate analysis.

**Table 4:** Univariate and multivariate ordinal logistic regression analyses for predictors of radiation-induced change (RIC) grade after GKRS.

	Univariate Analysis			Multivariate Analysis		
Factors	OR	CI	*p*-Value	OR	CI	*p*-Value
Sex (female)	1.046	(0.594, 1.842)	0.8766			
Age	1.012	(0.996, 1.029)	0.1450			
Target volume	1.061	(1.032, 1.091)	**<0.0001**			
Marginal dose	0.773	(0.622, 0.960)	**0.0198**			
Deep location	0.522	(0.247, 1.104)	0.0891			
Initial rupture	0.336	(0.184, 0.615)	**0.0004**	0.217	(0.104, 0.455)	**<0.0001**
SM grade	2.433	(1.678, 3.528)	**<0.0001**			
VRAS	1.753	(1.302, 2.360)	**0.0002**			
Pollock–Flickinger score	1.816	(1.389, 2.375)	**<0.0001**			
Measured volume within 50% isodense	1.035	(1.013, 1.057)	**0.0016**			
Nidus volume within 50% isodense line	1.092	(1.036, 1.152)	**0.0011**	0.842	(0.738, 0.962)	**0.0114**
Parenchyma volume within 50% isodense line	1.056	(1.018, 1.095)	**0.0036**	1.095	(1.028, 1.165)	**0.0045**
Nidus/Measured volume within 50% isodense line	0.006	(<0.001, 0.052)	**<0.0001**			
Parenchyma/Measured volume within 50% isodense line	169.033	(19.408, >999.999)	**<0.0001**			

OR = Odd ratio, CI = confidence interval, VRAS = Virginia Radiosurgery AVM Scale, GKRS = gamma knife radiosurgery, SM = Spetzler–Martin. Boldface type indicates statistical significance (*p* < 0.05). Grading scales were not included in the multivariate analysis.

## Data Availability

Data are available upon reasonable request.

## References

[1] Lunsford L.D., Kondziolka D., Flickinger J.C., Bissonette D.J., Jungreis C.A., Maitz A.H., Horton J.A., Coffey R.J. (1991). Stereotactic radiosurgery for arteriovenous malformations of the brain. J. Neurosurg..

[2] Pan D.H., Guo W.Y., Chung W.Y., Shiau C.Y., Chang Y.C., Wang L.W. (2000). Gamma knife radiosurgery as a single treatment modality for large cerebral arteriovenous malformations. J. Neurosurg..

[3] Inoue H.K. (2006). Long-term results of Gamma Knife surgery for arteriovenous malformations: 10- to 15-year follow up in patients treated with lower doses. J. Neurosurg..

[4] Flickinger J.C., Kondziolka D., Lunsford L.D., Pollock B.E., Yamamoto M., Gorman D.A., Schomberg P.J., Sneed P., Larson D., Smith V. (1999). A multi-institutional analysis of complication outcomes after arteriovenous malformation radiosurgery. Int. J. Radiat. Oncol. Biol. Phys..

[5] Izawa M., Hayashi M., Chernov M., Nakaya K., Ochiai T., Murata N., Takasu Y., Kubo O., Hori T., Takakura K. (2005). Long-term complications after gamma knife surgery for arteriovenous malformations. J. Neurosurg..

[6] Yamamoto M., Hara M., Ide M., Ono Y., Jimbo M., Saito I. (1998). Radiation-related adverse effects observed on neuro-imaging several years after radiosurgery for cerebral arteriovenous malformations. Surg. Neurol..

[7] Yen C.P., Matsumoto J.A., Wintermark M., Schwyzer L., Evans A.J., Jensen M.E., Shaffrey M.E., Sheehan J.P. (2013). Radiation-induced imaging changes following Gamma Knife surgery for cerebral arteriovenous malformations. J. Neurosurg..

[8] Serrato-Avila J.L., da Costa M.D.S., Stávale J.N., Lima J.V.F., Carrasco-Hernandez J.P., Alejandro S.A., Chaddad-Neto F. (2020). Microsurgical Resection of a Left Supramarginal Gyrus AVM Causing Radionecrosis. World Neurosurg..

[9] Ding D., Starke R.M., Sheehan J.P. (2017). Radiosurgery for the management of cerebral arteriovenous malformations. Handb. Clin. Neurol..

[10] Kano H., Flickinger J.C., Tonetti D., Hsu A., Yang H.C., Flannery T.J., Niranjan A., Lunsford L.D. (2017). Estimating the Risks of Adverse Radiation Effects After Gamma Knife Radiosurgery for Arteriovenous Malformations. Stroke.

[11] Flickinger J.C., Kondziolka D., Lunsford L.D., Kassam A., Phuong L.K., Liscak R., Pollock B. (2000). Development of a model to predict permanent symptomatic postradiosurgery injury for arteriovenous malformation patients. Arteriovenous Malformation Radiosurgery Study Group. Int. J. Radiat. Oncol. Biol. Phys..

[12] Flickinger J.C., Kondziolka D., Pollock B.E., Maitz A.H., Lunsford L.D. (1997). Complications from arteriovenous malformation radiosurgery: Multivariate analysis and risk modeling. Int. J. Radiat. Oncol. Biol. Phys..

[13] Flickinger J.C., Lunsford L.D., Kondziolka D., Maitz A.H., Epstein A.H., Simons S.R., Wu A. (1992). Radiosurgery and brain tolerance: An analysis of neurodiagnostic imaging changes after gamma knife radiosurgery for arteriovenous malformations. Int. J. Radiat. Oncol. Biol. Phys..

[14] Ilyas A., Chen C.J., Ding D., Buell T.J., Raper D.M.S., Lee C.C., Xu Z., Sheehan J.P. (2018). Radiation-Induced Changes After Stereotactic Radiosurgery for Brain Arteriovenous Malformations: A Systematic Review and Meta-Analysis. Neurosurgery.

[15] Chen C.J., Kearns K.N., Ding D., Kano H., Mathieu D., Kondziolka D., Feliciano C., Rodriguez-Mercado R., Grills I.S., Barnett G.H. (2019). Stereotactic radiosurgery for arteriovenous malformations of the basal ganglia and thalamus: An international multicenter study. J. Neurosurg..

[16] Ganz J.C., Reda W.A., Abdelkarim K. (2009). Adverse radiation effects after Gamma Knife Surgery in relation to dose and volume. Acta Neurochir. (Wien.).

[17] Han J.H., Kim D.G., Chung H.T., Park C.K., Paek S.H., Kim J.E., Jung H.W., Han D.H. (2008). Clinical and neuroimaging outcome of cerebral arteriovenous malformations after Gamma Knife surgery: Analysis of the radiation injury rate depending on the arteriovenous malformation volume. J. Neurosurg..

[18] Liscak R., Vladyka V., Simonova G., Urgosik D., Novotny J., Janouskova L., Vymazal J. (2007). Arteriovenous malformations after Leksell gamma knife radiosurgery: Rate of obliteration and complications. Neurosurgery.

[19] Tuleasca C., Peciu-Florianu I., Leroy H.A., Vermandel M., Faouzi M., Reyns N. (2020). Biologically effective dose and prediction of obliteration of unruptured arteriovenous malformations treated by upfront Gamma Knife radiosurgery: A series of 149 consecutive cases. J. Neurosurg..

[20] Peng S.J., Lee C.C., Wu H.M., Lin C.J., Shiau C.Y., Guo W.Y., Pan D.H., Liu K.D., Chung W.Y., Yang H.C. (2019). Fully automated tissue segmentation of the prescription isodose region delineated through the Gamma knife plan for cerebral arteriovenous malformation (AVM) using fuzzy C-means (FCM) clustering. Neuroimage Clin..

[21] Ganz J.C., Reda W.A., Abdelkarim K., Hafez A. (2005). A simple method for predicting imaging-based complications following gamma knife surgery for cerebral arteriovenous malformations. J. Neurosurg..

[22] Hayhurst C., Monsalves E., van Prooijen M., Cusimano M., Tsao M., Menard C., Kulkarni A.V., Schwartz M., Zadeh G. (2012). Pretreatment predictors of adverse radiation effects after radiosurgery for arteriovenous malformation. Int. J. Radiat. Oncol. Biol. Phys..

[23] Kim M.J., Park S.H., Park K.Y., Jung H.H., Chang J.H., Chang J.W., Lee J.W., Chang W.S. (2020). Gamma Knife Radiosurgery Followed by Flow-Reductive Embolization for Ruptured Arteriovenous Malformation. J. Clin. Med..

[24] Lee C.C., Chen C.J., Ball B., Schlesinger D., Xu Z., Yen C.P., Sheehan J. (2015). Stereotactic radiosurgery for arteriovenous malformations after Onyx embolization: A case-control study. J. Neurosurg..

[25] Hung Y.C., Mohammed N., Eluvathingal Muttikkal T.J., Kearns K.N., Li C.E., Narayan A., Schlesinger D., Xu Z., Sheehan J.P. (2019). The impact of preradiosurgery embolization on intracranial arteriovenous malformations: A matched cohort analysis based on de novo lesion volume. J. Neurosurg..

[26] Todnem N., Ward A., Nahhas M., Vender J.R., Alleyne C.H., Rahimi S.Y. (2019). A Retrospective Cohort Analysis of Hemorrhagic Arteriovenous Malformations Treated with Combined Endovascular Embolization and Gamma Knife Stereotactic Radiosurgery. World Neurosurg..

[27] Blamek S., Tarnawski R., Miszczyk L. (2011). Linac-based stereotactic radiosurgery for brain arteriovenous malformations. Clin. Oncol. (R. Coll. Radiol.).

[28] Inoue H.K., Kohga H., Hirato M., Nakamura M., Ohye C. (1994). Neurobiologic effects of radiosurgery: Histologic, immunohistochemical and electron-microscopic studies of a rat model. Stereotact. Funct. Neurosurg..

[29] Cohen-Inbar O., Lee C.C., Xu Z., Schlesinger D., Sheehan J.P. (2015). A quantitative analysis of adverse radiation effects following Gamma Knife radiosurgery for arteriovenous malformations. J. Neurosurg..

[30] Flickinger J.C., Kondziolka D., Maitz A.H., Lunsford L.D. (2002). An analysis of the dose-response for arteriovenous malformation radiosurgery and other factors affecting obliteration. Radiother Oncol..

[31] Flickinger J.C., Kondziolka D., Maitz A.H., Lunsford L.D. (1998). Analysis of neurological sequelae from radiosurgery of arteriovenous malformations: How location affects outcome. Int. J. Radiat. Oncol. Biol. Phys..

[32] Levegrün S., Hof H., Essig M., Schlegel W., Debus J. (2004). Radiation-induced changes of brain tissue after radiosurgery in patients with arteriovenous malformations: Correlation with dose distribution parameters. Int. J. Radiat. Oncol. Biol. Phys..

[33] Ganz J.C., Reda W.A. (2011). Radionecrosis following Gamma Knife treatment for mesial temporal lobe epilepsy. Br. J. Neurosurg..

[34] Lee C.C., Yang H.C., Lin C.J., Chen C.J., Wu H.M., Shiau C.Y., Guo W.Y., Hung-Chi Pan D., Liu K.D., Chung W.Y. (2019). Intervening Nidal Brain Parenchyma and Risk of Radiation-Induced Changes After Radiosurgery for Brain Arteriovenous Malformation: A Study Using an Unsupervised Machine Learning Algorithm. World Neurosurg..

[35] Ding D., Yen C.P., Xu Z., Starke R.M., Sheehan J.P. (2014). Radiosurgery for low-grade intracranial arteriovenous malformations. J. Neurosurg..

[36] Quigg M., Yen C.P., Chatman M., Quigg A.H., McNeill I.T., Przybylowski C.J., Yan G., Sheehan J.P. (2012). Risks of history of diabetes mellitus, hypertension, and other factors related to radiation-induced changes following Gamma Knife surgery for cerebral arteriovenous malformations. J. Neurosurg..

[37] Ding D., Yen C.P., Starke R.M., Xu Z., Sheehan J.P. (2015). Effect of prior hemorrhage on intracranial arteriovenous malformation radiosurgery outcomes. Cereb. Dis..

[38] Mohr J.P., Parides M.K., Stapf C., Moquete E., Moy C.S., Overbey J.R., Al-Shahi Salman R., Vicaut E., Young W.L., Houdart E. (2014). Medical management with or without interventional therapy for unruptured brain arteriovenous malformations (ARUBA): A multicentre, non-blinded, randomised trial. Lancet.

[39] Wedderburn C.J., van Beijnum J., Bhattacharya J.J., Counsell C.E., Papanastassiou V., Ritchie V., Roberts R.C., Sellar R.J., Warlow C.P., Al-Shahi Salman R. (2008). Outcome after interventional or conservative management of unruptured brain arteriovenous malformations: A prospective, population-based cohort study. Lancet Neurol..

[40] Al-Shahi Salman R., White P.M., Counsell C.E., du Plessis J., van Beijnum J., Josephson C.B., Wilkinson T., Wedderburn C.J., Chandy Z., St George E.J. (2014). Outcome after conservative management or intervention for unruptured brain arteriovenous malformations. Jama.

